# Supplementary material for: Fundamental niche unfilling and potential invasion risk of the slider turtle Trachemys scripta
Source: PeerJ. 2019 Oct 17;7:e7923. doi: 10.7717/peerj.7923 (PMC6800977; doi:10.7717/peerj.7923)
Supplement: Figure S1 [file peerj-07-7923-s001.docx]

**Fundamental niche unfilling and potential invasion risk of the slider turtle *Trachemys scripta***

Sayra Espindola, Juan L. Parra, Ella Vázquez-Domínguez

Supplemental Figure S1

**
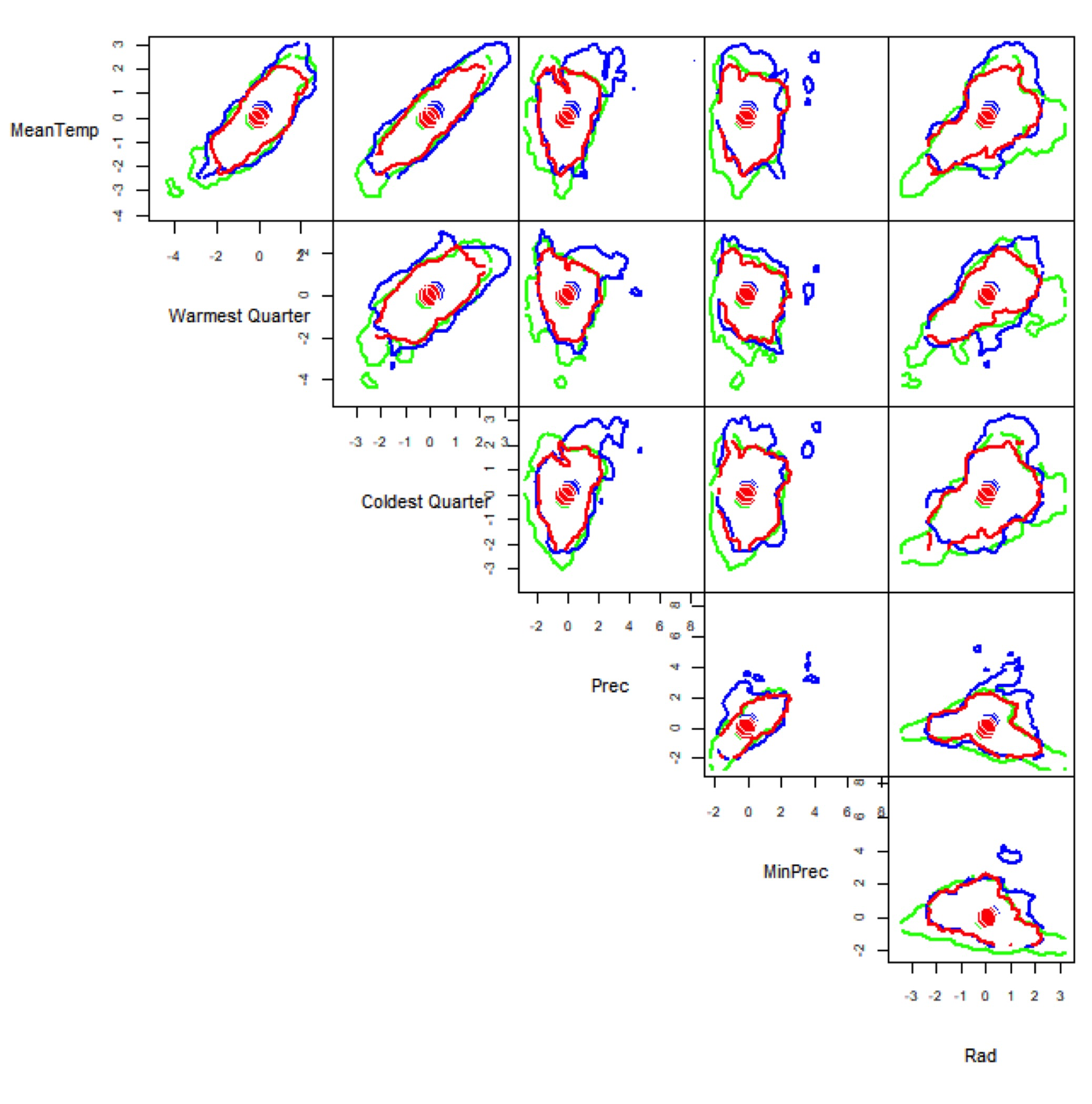
**

**Figure S1. Six-dimensional hypervolumes for native and non-native *Trachemys scripta*.** Estimated six-dimensional hypervolumes for native (green line), non-native (blue line), and intersection (red line) between *Trachemys scripta* native and non-native niches along the climatic space (Hypervolume package in R; Blonder *et al.* 2014). Variables were selected on the basis of the slider turtle’s natural history, and z-transformed for the analysis. Contours include 95% of simulated data.
