# Supplementary material for: Fundamental niche unfilling and potential invasion risk of the slider turtle Trachemys scripta
Source: PeerJ. 2019 Oct 17;7:e7923. doi: 10.7717/peerj.7923 (PMC6800977; doi:10.7717/peerj.7923)
Supplement: Supplemental Information 2 — Table S1. Literature review for T. scripta physiological optimums and tolerance limits. Table S2. Comparison between climatic covariance matrices for Trachemys scripta. Table S3. Contribution of the evaluated variables to the total hypervolume differences. [file peerj-07-7923-s002.docx]

**Fundamental niche unfilling and potential invasion risk of the slider turtle *Trachemys scripta***

Sayra Espindola, Juan L. Parra, Ella Vázquez-Domínguez

Supplemental Tables S2 – S4

**Table S1.** Literature review from where information about occurrence records, physiological optimums and tolerance limits for *Trachemys scripta* was obtained. Complete references are listed at the bottom.

| **Title** | **Year** | **Ref.** |
| --- | --- | --- |
| The growth of the Slider turtle, *Pseudemys scripta elegans.* | 1946 | 1 |
| Acute and chronic temperature effects on cardiovascular regulation in the red‑eared slider (*Trachemys scripta*). | 2015 | 2 |
| Varying Hydric Conditions during Incubation Influence Egg Water Exchange and Hatchling Phenotype in the Red‐Eared Slider Turtle. | 2008 | 3 |
| Anoxia tolerance and freeze tolerance in hatchling turtles. | 2005 | 4 |
| *Trachemys scripta* (Slider terrapin). | 2012 | 5 |
| An experimental study of the influence of embryonic water availability, body size, and clutch on survivorship of neonatal red-eared sliders, *Trachemys scripta elegans*. | 2002 | 6 |
| Basking Behavior of the Turtle *Pseudemys scripta*: Effects of Digestive State, Acclimation Temperature, Sex, and Season. | 1988 | 7 |
| Dietary and Habitat Shift with Size of Red-Eared Turtles (*Pseudemys scripta*) in a Southern Louisiana Population. | 1983 | 8 |
| Critical Thermal Maxima in Turtles. | 1966 | 9 |
| Experimental Test of the Effects of Fluctuating Incubation Temperatures on Hatchling Phenotype. | 2007 | 10 |
| Living at Extremes: Development at the Edges of Viable Temperature under Constant and Fluctuating Conditions. | 2009 | 11 |
| Acute and persistent effects of pre- and posthatching thermal environments on growth and metabolism in the red-eared slider turtle, *Trachemys scripta elegans*. | 2012 | 12 |
| Preferred body temperatures in five neartic freshwater turtles: a preliminary study. | 1993 | 13 |
| Cold Tolerance in Hatchling Slider Turtles (*Trachemys scripta*). | 1997 | 14 |
| Temperature, phenotype, and the evolution of temperature-dependent sex determination: how do natural incubations compare to laboratory incubations? | 2010 | 15 |
| Immigration and Dispersal of Slider Turtles *Pseudemys scripta* in Mississippi Farm Ponds. | 1984 | 16 |
| Basking Behavior of Emydid Turtles (*Chysemys picta, Graptemys geographica*, and *Trachemys scripta*) in an Urban Landscape. | 2009 | 17 |
| Effects of body temperature on righting performance of native and invasive freshwater turtles: Consequences for competition. | 2012 | 18 |
| Feeding status and basking requirements of freshwater turtles in an invasion context. | 2012 | 19 |
| Translating natural history into geographic space: a macroecological perspective on the North American Slider, *Trachemys scripta* (Reptilia, Cryptodira, Emydidae). | 2009 | 20 |
| Sexual and Seasonal Differences in Behavior of *Trachemys scripta* (Testudines: Emydidae). | 1999 | 21 |
| Response of Red-Eared Slider, *Trachemys scripta elegans*, Eggs to Slightly Differing Water Potentials. | 1998 | 22 |
| Year-to-Year Variation in Growth in the Red-Eared Turtle, *Trachemys scripta elegans*. | 1995 | 23 |
| Natural history notes on nesting, nests, and hatchling emergence in the red-eared slider turtle, *Trachemys scripta elegans*, in west central Illinois. | 1997 | 24 |
| Annual and Local Variation in Reproduction in the Red-Eared Slider, *Trachemys scripta elegans*. | 1998 | 25 |
| Temperature-Dependent Sex Determination in the Red-Eared Slider Turtle, *Trachemys scripta*. | 1998 | 26 |
| Linking climate and physiology at the population level for a key life-history stage of turtles. | 2005 | 27 |
| Temperature, Genes, and Sex: a Comparative View of Sex Determination in *Trachemys scripta* and *Mus musculus*. | 2005 | 28 |

1. Cagle F.R. 1946. The Growth of the Slider Turtle, *Pseudemys scripta elegans*. The American Midland Naturalist, 36, (3): 685-729.
2. Crossley II D.A., Wearng O.H., Platzacks B., Hartzler L.K., Hicks J.W. 2015. Acute and chronic temperature effects on cardiovascular regulation in the red‑eared slider (*Trachemys scripta*). J Comp Physiol B, 185:401–411.
3. Delmas V., Bonnet X., Girondot M., Pévot-Julliard A-C. 2008. Varying Hydric Conditions during Incubation Influence Egg Water Exchange and Hatchling Phenotype in the Red‐Eared Slider Turtle. Physiological and Biochemical Zoology: Ecological and Evolutionary Approaches, 81 (3): 345-355.
4. Dinkelacker S.A., Costanzo J.P., Lee Jr R.E. 2005. Anoxia tolerance and freeze tolerance in hatchling turtles. J Comp Physiol B, 175: 209-217.
5. Ficetola G.F., Rödder D. & Padoa-Schioppa E. 2012. *Trachemys scripta* (Slider terrapin). In: *Handbook of global freshwater invasive species* (ed. Francis R). Earthscan, Taylor & Francis Group Abingdon, UK, pp. 331-339.
6. Filoramo N.I., Janzen F.J. 2002. An experimental study of the influence of embryonic water availability, body size, and clutch on survivorship of neonatal red-eared sliders, *Trachemys scripta elegans*. Herpetologica, 58 (1): 67-74.
7. Hammond K.A., Spotila J.R., Standora E.A. 1988. Basking Behavior of the Turtle *Pseudemys scripta*: Effects of Digestive State, Acclimation Temperature, Sex, and Season. Physiological Zoology, 61 (1): 69-77.
8. Hart D.R. 1983. Dietary and Habitat Shift with Size of Red-Eared Turtles (*Pseudemys scripta*) in a Southern Louisiana Population. Herpetologica, 39 (3):285-290.
9. Hutchison V.H., Vinegar A., Kosh R.J. 1966. Critical Thermal Maxima in Turtles. Herpetologica, 22(1):32-41.
10. Les H.L., Paitz R.T., Bowden R.M. 2007. Experimental Test of the Effects of Fluctuating Incubation Temperatures on Hatchling Phenotype. Journal of Experimental Zoology, 307A: 274-280.
11. Les H.L., Paitz R.T., Bowden R.M. 2009. Living at Extremes: Development at the Edges of Viable Temperature under Constant and Fluctuating Conditions. Physiological and Biochemical Zoology, 82 (2): 105-112.
12. Ligon D.B., Peterson C.C., Lovern M.B. 2012. Acute and persistent effects of pre- and posthatching thermal environments on growth and metabolism in the red-eared slider turtle, *Trachemys scripta elegans*. J. Exp. Zool. 317:227–235.
13. Nutting W.L., Graham T.E. 1993. Preferred body temperatures in five neartic freshwater turtles: a preliminary study. Comp. Biochem. Physiol., 104A (2): 243-246.
14. Packard G.C., Tucker J.K. 1997. Cold Tolerance in Hatchling Slider Turtles (*Trachemys scripta*). Copeia, 1997 (2): 339-345.
15. Paitz R.T., Gould A.C., Holgersson M.C.N., Bowden R.M. 2010. Temperature, phenotype, and the evolution of temperature-dependent sex determination: how do natural incubations compare to laboratory incubations? J. Exp. Zool. (Mol. Dev. Evol.) 314B:86–93.
16. Parker W.S. 1984. Immigration and Dispersal of Slider Turtles *Pseudemys scripta* in Mississippi Farm Ponds. The American Midland Naturalist, 112(2): 280-293.
17. Peterman W.E., Ryan T.J. 2009. Basking Behavior of Emydid Turtles (*Chysemys picta, Graptemys geographica*, and *Trachemys scripta*) in an Urban Landscape. Northeastern Naturalist, 16 (4): 629-636.
18. Polo-Cavia N., López P., Martín J. 2012. Effects of body temperature on righting performance of native and invasive freshwater turtles: Consequences for competition. Physiology & Behavior, 108: 28-33.
19. Polo-Cavia N., López P., Martín J. 2012. Feeding status and basking requirements of freshwater turtles in an invasion context. Physiology & Behavior 105: 1208–1213.
20. Rodder D., Kwet A., Lotters S. 2009. Translating natural history into geographic space: a macroecological perspective on the North American Slider, *Trachemys scripta* (Reptilia, Cryptodira, Emydidae). Journal of Natural History, 43 (39-40): 2525-2536.
21. Thomas R.B., Vogrin N., Altig R. 1999. Sexual and Seasonal Differences in Behavior of *Trachemys scripta* (Testudines: Emydidae). Journal of Herpetology, 33 (3): 511-515.
22. Tucker J.K., Filoramo N.I., PAukstis G.L., Janzen F.J. 1998. Response of Red-Eared Slider, *Trachemys scripta elegans*, Eggs to Slightly Differing Water Potentials. Journal of Herpetology, 32 (1):124-128.
23. Tucker J.K., Maher R.J., Theiling C.H. 1995.Year-to-Year Variation in Growth in the Red-Eared Turtle, *Trachemys scripta elegans*. Herpetologica, 51(3):354-358.
24. Tucker, J. K. 1997. Natural history notes on nesting, nests, and hatchling emergence in the red-eared slider turtle, *Trachemys scripta elegans*, in west central Illinois. Illinois Natural History Survey Biological Notes 140. 13 pp.
25. Tucker, J.K., Paukstis G.L., Janzen F.J. 1998. Annual and Local Variation in Reproduction in the Red-Eared Slider, *Trachemys scripta elegans*. Journal of Herpetology, 32(4):515-526.
26. Wibbels T., Cowan J., LeBoeuf R. 1998. Temperature-Dependent Sex Determination in the Red-Eared Slider Turtle, *Trachemys scripta*. Journal of Experimental Zoology, 281: 409-416.
27. Willette A.S., Tucker J.K., Janzen F.J. 2005. Linking climate and physiology at the population level for a key life-history stage of turtles. Can. J. Zool., 83: 845-850.
28. Yao H. H-C., Capel B. 2005. Temperature, Genes, and Sex: a Comparative View of Sex Determination in *Trachemys scripta* and *Mus musculus*. J. Biochem., 138: 5-12.

**Table S2.** Comparison between climatic covariance matrices for *Trachemys scripta*, showing their corresponding Akaike information criterion (AIC) values. (Step-up and model building approaches).

| **Model** | | **AIC** |
| --- | --- | --- |
| **higher** | **lower** |  |
| Equality | Proportional | 6634.758 |
| Proportional | CPC | 2443.042 |
| CPC | CPC(4) | 1710.512 |
| CPC(4) | CPC(3) | 1587.103 |
| CPC(3) | CPC(2) | 1509.213 |
| CPC(2) | CPC(1) | 1205.078 |
| CPC(1) | Unrelated | 732.403 |
| Unrelated | --- | 42.000 |

**Table S3.** Contribution of the evaluated variables to the total hypervolume differences between *Trachemys scripta* native and non-native niches. The importance score reported is the ratio of the n-dimensional hypervolume relative to each of the n-1 dimensional hypervolumes, where larger values indicate that a variable contributes proportionally more to the overall volume.

| **Variable** | **Native occupied niche** | **Non-native occupied niche** |
| --- | --- | --- |
| **bio01** | 0.9686791 | 0.9576779 |
| **bio10** | 1.1684712 | 1.1302481 |
| **bio11** | 1.0670855 | 0.992571 |
| **bio12** | 1.1333105 | 1.2999334 |
| **bio14** | 1.152466 | 1.1045099 |
| **radAnual** | 1.1497316 | 1.110255 |
