# Supplementary material for: Fundamental niche unfilling and potential invasion risk of the slider turtle Trachemys scripta
Source: PeerJ. 2019 Oct 17;7:e7923. doi: 10.7717/peerj.7923 (PMC6800977; doi:10.7717/peerj.7923)
Supplement: Glossary S1 — Definition of some main terms used in the manuscript. [file peerj-07-7923-s003.docx]

**Fundamental niche unfilling and potential invasion risk of the slider turtle *Trachemys scripta***

Sayra Espindola, Juan L. Parra, Ella Vázquez-Domínguez

Supplemental Glossary S1

**Glossary**

**Analog climate**: the combination of climatic conditions present in one area (or time) that is within the envelope of climatic conditions found in a different area (or time) and used for comparison. The contrary is referred to as **non-analog climate**.

**Environmental space**: all the possible combinations between two environmental variables (or more in a *n*-multidimensional space), which can be represented –or not– on the geographic space; also referred to as “environmental range”. In Figure 1, the environmental space is delimited by the blue square box (“a”).

**Fundamental niche**: of a species consisting of the suite of environmental (abiotic) conditions in a *n*-dimensional environmental space that permit survival and reproduction of individuals (Hutchinson, 1978). It is determined by an organism’s physiological range of tolerance to environmental factors where a species can survive in the absence of biotic interactions (Soberón & Arroyo-Peña, 2017). Hence, the physiological requirements of a species (i.e. optimum, minimum and maximum physiological tolerance limits) can be measured to delimit the dimensions of its fundamental niche. In Figure 1, “d” depicts the fundamental niche; notice that it also refers to areas of the fundamental niche that are not currently available on the geographic space.

**Native range**: the complete geographic area where a species is naturally distributed (where an exotic species is native), whereas **Non-native range** is the geographic area where an exotic (non-native) species is distributed outside its native range. In Figure 1 “b” and “c” depict the environment available at the native and non-native ranges, respectively.

**Niche expansion**: proportion of the exotic niche that does not overlap with the native niche.

**Niche overlap**: the intersection of two niches in the *n*-dimensional environmental space.

**Niche shift**: a change in the limits of the niche envelope in environmental space.

**Niche stability**: proportion of the non-native niche overlapping with the native niche.

**Niche unfilling**: proportion of the native niche that does not overlap with the non-native niche.

**Potential native distribution**: suitable areas inside the native geographic range (occupied or not); also referred to as “potential native range”.

**Potential non-native distribution**: suitable areas outside the native geographic range (occupied or not); also referred to as “potential non-native range”.

**Realized niche**: it is the actual area occupied by a species (quantified from field observations and occurrence records); also referred to as “ecological niche”; usually a more restricted region of the environmental (abiotic) conditions obtained after accounting for biotic interactions, populations dynamics and dispersal limitations. It comprises a subset of the fundamental niche, which is constrained by biotic factors and may prevent individuals of a species from occupying part of its fundamental niche. The geographic projection of the realized niche is based on the correlation between occurrence records and bioclimatic variables. In Figure 1, “e” and “f” depict the realized (occupied) niche at the native and non-native ranges, respectively.

Hutchinson, G.E. (1957). Concluding remarks. Cold Spring Harbor Symposia on Quantitative Biology, 22, 425-427.

Hutchinson, G.E. (1978). An introduction to population ecology. Yale University Press, New Haven.

Jackson, S.T., & Overpeck, J.T. (2000). Responses of plant populations and communities to environmental changes of the late Quaternary. *Paleobiology*, 26, 194-220.

Kearney, M., & Porter, W. (2009). Mechanistic niche modelling: combining physiological and spatial data to predict species’ ranges. *Ecology Letters*, 12, 334-350.

Kearney, M., Phillips, B.L., Tracy, C.R., Christian, K.A., Betts, G., & Porter, W.P. (2008). Modelling species distributions without using species distributions: the cane toad in Australia under current and future climates. *Ecography*, 31, 423-434.

Peterson A.T., Soberón, J., Pearson, R.G., Anderson, R.P., Martínez-Meyer, E., Nakamura, M., & Araújo, M.B. (2011). Ecological Niches and Geographic Distributions. Princeton University Press.

Soberón, J. (2007). Grinnellian and Eltonian niches and geographic distributions of species. *Ecology Letters*, 10, 1115-1123.

Soberón, J., & Arroyo-Peña (2017).

Tingley, R., Vallinoto, M., Sequeira, F., & Kearney, M.R. (2014). Realized niche shift during a global biological invasion. *Proceedings of the National Academy of Sciences, USA*, 111, 10233-10238.
